# “Tote Fair,” Doctor—It Doesn’t Pay the Other Way

**Published:** 1911-08

**Authors:** 


### "Tote Fair," Doctor—It Doesn't Pay the Other Way.

Many men are tempted in a lifetime to take the short cuts; some do it only once, and get enough in the start, while others live entirely by such methods, and finally learn, when too late, that it is a losing game every time—the greatest loss being that of principle, for when a man sinks in his own estimation, he is low indeed.

Some men resist temptation feebly, and for such that certain clause in the decalogue was evidently placed by one who understood human nature as it would develop all along the march of time. It is perfectly absurd to our mind that our Creator would ever lead us into temptation, whether we prayed or not. It is not in keeping with the attributes of one so just and loving. But we are equally certain that many of us poor weaklings are frequently lead into temptation by agents a long way this side of the heavenly throne.

We have either dreamed, read or heard it said that every man has his price; that he only needs sufficient temptation to make him prove traitor to principle, and stain his hands in conscience blood. When we apply this to the profession in its relation to its own members we must admit that there is but small temptation to steal a pauper patient from a medical brother, though, where the habit is strong, and the resistance very feeble, we have known this to be done, as an act of pure malignity, and just to "keep the fellow's hand in." A pauper can talk and lie, and frequently he can be persuaded to give his real benefactor a professional black eye, while talking some medical sneak into better practice. Therefore, the clean-handed physician can not always be dead sure of his charity practice, even if they can be patched up and used as decoys for better game by unscrupulous men who wear the livery of the profession. Now, when it comes to big game—the patient who is well heeled, gilded, whose money is plentiful and good, and whose influence is still better—we just tremble for the man who has him; for he must hustle to keep him. If there ever is a supreme test of principle in the life of the medical half-breed, it comes when he has an inning at a wealthy and influential patient that rightfully belongs to another physician. When, by a little half-excusable crooked work, he can oust the attendant and step in his shoes, unless the blue blood is there he will "break shot" in spite of the devil, as the sportsman says.

We have seen all of this—seen the curs and mongrels pile over each other, and, after consuming the quarry, cut their own throats; and we have also seen the thoroughbred stand while the mongrels flushed the game from under his nose, and continue to stand until every muscle grew rigid and tense in his victory of principle over temptation. Readers, we are not talking about dogs; we are thinking about doctors, and we wish to say to certain doctors, some of whom may be in our own town, that when a gentleman sends a specialist a patient under his own treatment, relying upon his honor to treat him and the patient squarely, and when that same specialist works this patient away from the physician who sent him to him, and gets him into the hands of another medical man, he has "broken shot" and lost caste. Doctor, it will not pay to be a mongrel.—Editorial in *Southern Clinic,* Richmond, Va., July, 1911.

